# Protocol for a national monthly survey of alcohol use in England with 6-month follow-up: ‘The Alcohol Toolkit Study’

**DOI:** 10.1186/s12889-015-1542-7

**Published:** 2015-03-07

**Authors:** Emma Beard, Jamie Brown, Robert West, Crispin Acton, Alan Brennan, Colin Drummond, Matthew Hickman, John Holmes, Eileen Kaner, Karen Lock, Matthew Walmsley, Susan Michie

**Affiliations:** Research Department of Clinical, Educational and Health Psychology, University College London, London, UK; Department of Epidemiology and Public Health, University College London, London, UK; Alcohol Misuse, Department of Health, London, UK; ScHARR, The University of Sheffield, Sheffield, UK; Institute of Psychiatry, Kings College London, London, UK; School of Social and Community Medicine, University of Bristol, Bristol, UK; Institute of Health & Society, Newcastle University, Newcastle, UK; Faculty of Public Health and Policy, London School of Hygiene and Tropical Medicine, London, UK; Public Health England, London, UK

**Keywords:** Alcohol toolkit study, Epidemiology, Smoking toolkit study, Alcohol consumption, AUDIT

## Abstract

**Background:**

Timely tracking of national patterns of alcohol consumption is needed to inform and evaluate strategies and policies aimed at reducing alcohol-related harm. Between 2014 until at least 2017, the Alcohol Toolkit Study (ATS) will provide such tracking data and link these with policy changes and campaigns. By virtue of its connection with the ‘Smoking Toolkit Study’ (STS), links will also be examined between alcohol and smoking-related behaviour.

**Methods/Design:**

The ATS consists of cross-sectional household, computer-assisted interviews of representative samples of adults in England aged 16+. Each month a new sample of approximately 1800 adults complete the survey (~n = 21,600 per year). All respondents who consent to be followed-up are asked to complete a telephone survey 6 months later. The ATS has been funded to collect at least 36 waves of baseline and 6-month follow-up data across a period of 3 years. Questions cover alcohol consumption and related harm (AUDIT), socio-demographic characteristics, attempts to reduce or cease consumption and factors associated with this, and exposure to health professional advice on alcohol. The ATS complements the STS, which has been tracking key performance indicators relating to smoking since 2006. As both the ATS and STS involve the same respondents, it is possible to assess interactions between changes in alcohol and tobacco use. Data analysis will involve: 1) Descriptive and exploratory analyses undertaken according to a pre-defined set of principles while allowing scope for pursuing lines of enquiry that arise from prior analyses; 2) Hypothesis testing according to pre-specified, published analysis plans. Descriptive data on important trends will be published monthly on a dedicated website: www.alcoholinengland.info.

**Discussion:**

The Alcohol Toolkit Study will improve understanding of population level factors influencing alcohol consumption and be an important resource for policy evaluation and planning.

## Background

The UK has among the highest per capita alcohol consumption of any country in the world [1,2], with 9.1 million adults drinking at levels above recommended limits [3,4]. The costs to society in terms of the health, social and criminal implications of excessive alcohol consumption are estimated to be more than £21 billion per annum [5]. In 2010, 3.5% of all deaths in England were wholly attributable to excessive alcohol consumption [6,7]. A further 1.1% of deaths were partially attributable [6].

The rates of harmful drinking in a given country are a function of many factors, including financial cost, cultural norms [8-11] and beliefs about alcohol-related harm [3]. In 2012 the English Government’s alcohol strategy proposed a range of policies to tackle alcohol-related harm: 1) helping individuals to change their drinking behaviour; 2) taking action locally; 3) improving treatment for alcohol dependence; 4) sharing responsibility with the alcohol industry; 5) imposing a minimum unit price; and 6) extending restrictions on advertisement to teenagers and children [5]. Since then the Government has withdrawn some policies (e.g., minimum unit pricing) but has gone ahead with some others: a ban on sales of alcohol ‘below cost’; both reductions and increases in alcohol duties; introducing screening and brief intervention for risk drinking as part of NHS Health Checks; and voluntary agreements with industry to reduce availability of high-strength canned beverages, lower the strength of existing beverages and promote low strength alternatives, and increase the number of product labels providing alcohol content information [12-14]. Alcohol charities have also promoted ‘Dry January’ (www.dryjanuary.org.uk) to encourage drinkers to abstain for one month, while more than 70 local authorities have voluntary agreements with retailers to withdraw high-strength, low-cost beers and ciders from sale [15].

Timely and detailed surveillance data could help to inform and evaluate national and local alcohol policies. Several large-scale surveys collect data on alcohol use in the United Kingdom on an annual or less frequent basis [16] (e.g. Health Survey for England, General Lifestyle Survey, and Adult Psychiatric Morbidity Survey; See Table 1).

**Table 1:** **Topics addressed by surveys in England regarding alcohol use**

**Representative surveys**	**Outline**	**Sample**	**Design**	**Alcohol questions addressed at baseline**	**Additional notes**
National Diet and Nutrition Survey (NDNS) [17]	Survey of food consumption, nutrient intakes and nutritional status. Set up in 1992/93 following the 1986/87 Dietary and Nutritional Survey of British Adults [18]	1992 to 1999 surveys covered four age groups: pre-school children (aged 1.5 to 4.5 years), young people (aged four to 18 years), adults (aged 19 to 64 years) and older adults (aged 65 and over).	Initially consisted of cross-sectional surveys conducted every 3 years. Since 2008, surveys have been conducted annually with the addition of a “four-day food diary”.	• Alcohol consumption in the past week	Two year delay in publication of the findings.
				• On the heaviest drinking day, amount drunk	
				• Type of alcohol consumed	
		2000/2001 survey covered those aged 19 to 64 years.			
		2008 to present surveys cover all individuals’ aged 1.5 years and older living in private households. Data are currently collected on around 500 adults (aged 19 and over) and 500 children each year (aged 1.5 to 18 years).			
Understanding Society, the UK Household Longitudinal Study (UKHLS) [19]	Set up in 2009. Consists of a longitudinal survey of subject’s health, work, education, income, family, and social life.	Data collected on 40,000 households. Adult household members aged 16 or older are given the full length questionnaire and those aged 10–15 years of age are asked to complete a shorter version.	Panel survey of households with yearly interviews. Data collection for a single wave is scheduled across 24 months.	• Amount spent by the household on alcohol in the past month	One to two year delay in publication of the findings. Only amount spent regularly assessed.
				• Frequency of alcohol consumption whilst pregnant (ranged from less than one month to every day)	
				• Units of alcohol per week and per day	
				• Ever consumption of alcohol	
				• Number of alcoholic drinks in the past month	
				• Number of times intoxicated in the past month	
Opinions and Lifestyle Survey (OPN) [20]	Set up in 2012. A combination of the Opinions Survey and General Lifestyle Survey. Assesses individuals drinking, smoking and health, use of medical services, family information and fertility.	Data collected on 13,200 adults per year (aged 16+).	Monthly survey. Surveys do not run one month in every three years (June, September, December and March).	• Number of days drank in the previous week	One year delay in publication of the findings. Some questions from the General Lifestyle Survey were modified and so results may not be directly comparable (for further details see [21]). For example, the survey does not ask about the specific alcohol by volume (ABV) of every alcoholic drink but assumes an average for each type of drink.
				• On the heaviest drinking day, the types and amount of alcoholic drinks consumed	
Health Survey for England (HSE) [22]	Set up in 1991. Involves a series of surveys to measure health and health related behaviours in adults and children.	Data collected on 5000–15000 adults each year (aged 16+). Since 1995, it has also included children aged 2–15 years of age. Information on children under 13 is obtained from a parent.	Survey conducted annually.	• Frequency of drinking in the last 12 months (including those who never drink)	One year delay in publication of the findings. Until 1997, drinking was measured using a series of questions that, for each type of drink, recorded the frequency of drinking within the last 12 months and the usual amount drunk on any single day. These quantity-frequency questions were dropped in 2003, but reinstated in 2011. Questions on the amount drunk in the past week were introduced in 2008.
				• Number of drinking days in the last week	
				• For those who drank in the last week, the amounts of different types of alcohol drunk on the day they drank most.	
				• For those who drank in the last 12 months, the frequency of drinking different types of drink and the amounts of each drunk on a typical day.	
General Lifestyle Survey (GLF)/General Household Survey (GHS) [23].	Set up in 1971 and terminated in 2012, when it became part of the Opinions and Lifestyle Survey. Collects information on demographic and health information, covering a wide range of topics.	Data collected on 14,500 adults each year living in private households.	Surveys conducted annually with follow-ups for 4 years. Break in 1997–1998 and 1999–2000.	• Maximum amount drunk on any one day in the previous seven days	Two year delay in publication of the findings. Questions on alcohol consumption only available post 1986 from the GHS and from 2008 from the GLF
				• Average weekly alcohol consumption	
Adult Psychiatric Morbidity Survey (APMS) [24].	Set up in 1999. Collects data on the prevalence of both treated and untreated psychiatric disorders.	Data collected on 8,000 working age adults each year in private households. In 1993 data collected on 16–64 year olds; in 2000 on 16–74 year olds; and in 2007 on 16–74 year olds.	Surveys conducted every 7 years.	• Hazardous and harmful drinking measured by the AUDIT	Two year delay in publication of the findings.
				• Alcohol dependence measured by the SADQ-C	
Alcohol Toolkit study (ATS)	Set up in 2014. This survey assesses alcohol consumption and related parameters; and is based on and linked with the Smoking Toolkit Study (STS).	Data collected on 20,400 adults each year aged 16+.	Monthly cross sectional surveys with 6 months follow-up.	• Hazardous and harmful drinking measured by the AUDIT	No delay in publication. Data published monthly on www.alcoholinengland.info.
				• Current attempts to cut down consumption	
				• GP/health-care professional advice	
				• Type of alcohol consumed	
				• Motivation to cut down and consumption	
				• Amount spent	
				• Strength of urges to drink	
				• Number of recent serious attempts to cut down	
				• Aids used to help cut down	
				• Motives for attempts to cut down	
International Alcohol Control Policy Evaluation Study (IAC): England and Scotland study (APISE) [25]	Set up in 2012. This survey assesses alcohol consumption and related parameters.	Aims to collect data on 2000 adults (aged 16+) in England.	Annual survey with 12 month follow-up.	• Proximal measures of policy impact (e.g. for the impact of pricing: product selection, and amount purchased, priced paid and price salience).	Some delay in publication of the findings.
				• Frequency of drinking and typical occasion quantity	
				• Preloading	
				• Drinking location (e.g. pub, own home)	
				• Perceptions of availability and affordability	

Note: AUDIT: Alcohol Use Disorders Identification Test; SADQ-C: Severity of Alcohol Dependence Questionnaire; The questions are those which at the time of publication were asked in the surveys. Additional questions may be added or questions modified.

The ATS will fill an important gap by gathering and publishing monthly data on representative samples, including detailed information on alcohol use, attempts to reduce or cease alcohol consumption, and exposure to health professional advice on alcohol. It includes a 6-month telephone follow-up of each monthly sample which will provide data on within-individual trends and consistency in alcohol-related measures.

The ATS is modelled on, and involves the same respondents as the Smoking Toolkit Study (STS) [26], which was set up in 2006 and has already collected data on approximately 175,000 individuals. The STS has demonstrated the value of having monthly figures on key performance indicators and that it has the granularity to detect temporal trends that would otherwise be missed [27-31]. The linkage of these two surveys will provide a unique opportunity to compare trends in smoking and alcohol use behaviour at an individual, regional and national level, and to examine the interdependencies between these two behaviours.

### Aims

The ATS aims to provide timely tracking of alcohol-related behaviours on a monthly basis to inform and evaluate national alcohol control policies in England. It will also permit analysis of trends as a function of major socio-demographic variables. Monthly, quarterly and annual trends will be published (overall and stratified by age group, social grade, region, gender and smoking status) on:

1. Prevalence of hazardous drinking (Measured by the Alcohol Use Disorders Identification Test (AUDIT))
2. Prevalence of hazardous drinkers who report attempting to reduce their alcohol consumption
3. Prevalence of different methods used by hazardous drinkers attempting to reduce their consumption
4. Prevalence among hazardous drinkers of receipt of advice to reduce alcohol consumption from a health professional in the past year

Changes in these variables associated with events, such as the introduction of pricing policies, will also be assessed.

## Methods

### Design

The ATS involves monthly cross-sectional household computer-assisted interviews, conducted by Ipsos Mori, of approximately 1,800 adults aged 16+ and over in England. All participants who agree to be re-contacted are followed-up at 6 months by a telephone survey. Baseline data were first collected in March 2014, to be followed up in September 2014.

The baseline survey uses a type of random location sampling, which is a hybrid between random probability and simple quota sampling. England is first split into 171,356 ‘Output Areas’, comprising of approximately 300 households. These areas are then stratified according to ACORN characteristics and geographic region. ACORN is a socio-economic profiling tool developed by CACI (http://www.caci.co.uk/acorn/), and works by segmenting UK postcodes into 5 categorises (wealthy achievers, urban prosperity, comfortably off, moderate means and hard-pressed). These categorises are further divided into 17 groups and 56 types using government and consumer research data (e.g., census data and lifestyle survey records).

The areas are then randomly allocated to interviewers, who travel to their selected areas and conduct the electronic interviews with one member of the household. Interviews are conducted until quotas based upon factors influencing the probability of being at home (i.e. working status, age and gender) are fulfilled. Morning interviews are avoided to maximise participant availability.

This method of sampling is often seen as superior to conventional quota sampling because the choice of properties approached is significantly reduced by randomly allocating small output areas to the interviewers. However, as interviewers still choose the houses within these particular areas, a response rate cannot be calculated. This is because there is no definite *gross sample*, with units fulfilling the criteria of the quota being interchangeable.

Ethical approval for the STS was granted by the UCL Ethics Committee (ID 0498/001). Further ethical approval for the ATS was not deemed necessary by the Committee, since asking individuals about their alcohol consumption in addition to smoking does not place them at additional risk of harm.

### Sample

The study is initially funded for a three year period between March 2014 and March 2017. It is anticipated that data on around 21,600 individuals will be collected each year, with a proposed 3 year sample size of 64,800. However, as with the STS, which has been collecting data for eight years, it is envisaged that the ATS will be extended beyond this initial period.

### Measures

Questions were developed by an expert panel and policy-makers. The key domains addressed at baseline are: 1) prevalence and frequency of harmful alcohol consumption (AUDIT) [32]; 2) current attempts and motivation to reduce alcohol consumption below harmful levels; 3) health-care professional advice about alcohol consumption; 4) types of drinks consumed and amount spent; 4) urges to drink; 5) recent serious attempts to cut down; and 7) help sought and factors contributing to recent attempts to reduce intake. Additional questions, as with the STS, can be added if new hypotheses are generated. The STS and ATS were set up as ‘toolkits’ for researchers in alcohol and tobacco control, affording the ability to add specific questions (e.g. on mental health). Thus researchers would be provided with all the contextual measurements listed below, negating the requirement for multiple surveys.

### Baseline measures

#### Socio-demographic characteristics

Data are collected on gender, ethnicity, socio-economic status, marital status, number of residents and children in the household, sexuality, age, disability, religion, internet access and use, and government office region (London, South East, South West, East Anglia, East Midlands, West Midlands, Yorkshire/Humberside, North West and North East). Socio-economic status is assessed through car and home ownership, employment status, educational achievements, income and by social-grade. Social grade is measured using the National Readership Survey social-grades system: A: higher managerial, administrative or professional; B: intermediate managerial, administrative or professional; C1: supervisory or clerical and junior managerial administrative or professional; C2: skilled manual workers; D: Semi and unskilled manual workers; and E: Causal or lowest grade workers, pensioners and others who depend on the welfare state for their income [33].

#### Smoking-related questions

All participants taking part in the ATS are also asked questions regarding their use of tobacco products and smoking behaviour. Full details of these questions and details of the methodology are available from www.smokinginengland.info and Fidler et al. [26].

#### Prevalence and frequency of hazardous drinking

The AUDIT (See Table 2: Question 1: a-k) is used to screen for alcohol use [32]. The AUDIT identifies people who could be classed as dependent, harmful or hazardous drinkers; and has proven validity, high internal consistency and good test-retest reliability across gender, age and cultures [34-38]. The full AUDIT consists of 10 questions: questions 1–3 deal with alcohol consumption, 4–6 with alcohol dependence and 7–10 with alcohol-related problems. A score of 8 or more in men (sometimes 7 in women) indicates a hazardous or harmful alcohol consumption, whilst a score greater than 20 is suggestive of alcohol dependence. The ATS uses an extended item version of the AUDIT questionnaire adopted in previous ‘Screening and Brief Alcohol Intervention in Primary Care (SIPS)’ trials; to allow exploration of higher levels of alcohol consumption in addition to the standard AUDIT scoring system [39,40]. The 2009 Adult Psychiatric Morbidity Survey reported that 24.2% of participants scored 8 or above on the AUDIT, which included 3.8% of adults whose drinking could be classified as harmful. Among males, the highest prevalence of hazardous and harmful drinking was in the 25–34 age group; whereas in females it was in those aged 16–24 [41].

**Table 2:** **Main baseline questionnaire for the ATS**

**1. Audit**	
a. How often do you have a drink containing alcohol?	• Never
	• Monthly or less
	• 2 to 4 times a month
	• 2 to 3 times a week
	• 4 to 5 times a week
	• 6 or more times a week
b. How many standard drinks containing alcohol do you have on a typical day when you are drinking?	• 1 to 2
	• 3 to 4
	• 5 to 6
	• 7 to 9
	• 10 to 12
	• 13 to 15
	• 16 or more
c. How often do you have six or more standard drinks on one occasion?	• Never
	• Less than monthly
	• Monthly
	• Weekly
	• Daily or almost daily
d. How often during the last 6 months have you found that you were not able to stop drinking once you had started?	• Never
	• Less than monthly
	• Monthly
	• Weekly
	• Daily or almost daily
e. How often during the last 6 months have you failed to do what was normally expected from you because of drinking?	• Never
	• Less than monthly
	• Monthly
	• Weekly
	• Daily or almost daily
f. How often during the last 6 months have you needed a first drink in the morning to get yourself going after a heavy drinking session?	• Never
	• Less than monthly
	• Monthly
	• Weekly
	• Daily or almost daily
g. How often during the last 6 months have you had a feeling of guilt or remorse after drinking?	• Never
	• Less than monthly
	• Monthly
	• Weekly
	• Daily or almost daily
h. How often during the last 6 months have you been unable to remember what happened the night before because you had been drinking?	• Never
	• Less than monthly
	• Monthly
	• Weekly
	• Daily or almost daily
j. Have you or someone else ever been injured as a result of your drinking?	• No
	• Yes, but not in the least 6 months
	• Yes, during the last 6 months
Has a relative or friend or a doctor or another health worker ever been concerned about your drinking or suggested you cut down?	• No
	• Yes, but not in the least 6 months
	• Yes, during the last 6 months
**2. Current attempts to reduce intake**	
a. Are you currently trying to restrict your alcohol consumption e.g. by drinking less, choosing lower strength alcohol or using smaller glasses?	• Yes
	• No
**3. GP advice**	
a. In the last 12 months, has a doctor or other health worker within your GP surgery discussed your drinking?	• No
	• Yes, a doctor or other health worker within my GP surgery offered advice about cutting down on my drinking
	• Yes, a doctor or other health worker within my GP surgery offered help or support within the surgery to help me cut down
	• Yes, a doctor or other health worker within my GP surgery referred me to an alcohol service or advised me to seek specialist help
	• Yes, a doctor or other health worker within my GP surgery referred me to an alcohol service or advised me to seek specialist help
b. (If answers No) You said a doctor or other health worker within your GP surgery has not discussed your drinking with you in the last 12 months. Please could you confirm which of the following statements applies to you.	• I have not seen a doctor or health worker within my GP surgery in the last 12 months
	• I have seen a doctor or health worker within my GP surgery in the last 12 months but did not discuss my drinking.
**4. Type of alcohol**	
a. Which of these do you drink most often?	• Wine
	• Beer or lager
	• Spirits on their own (for example whisky, vodka)
	• Cider
	• Alcopops (for example WKD, Smirnoff Ice)
	• Mixed drinks (for example gin and tonic, whisky and coke)
	• Other
**5. Motivation to reduce**	
a. Which of the following best describes you?	• I REALLY want to cut down on drinking alcohol and intend to in the next month
	• I REALLY want to cut down on drinking alcohol and intend to in the next 3 months
	• I want to cut down on drinking alcohol and hope to soon
	• I REALLY want to cut down on drinking alcohol but I don’t know when I will
	• I want to cut down on drinking alcohol but haven’t thought about when
	• I think I should cut down on drinking alcohol but don’t really want to
	• I don’t want to cut down on drinking alcohol
**6. Amount spent**	
a. On average about how much per week do you think you spend on alcohol for your own consumption?	
**7. Urges to drink**	
a. How strongly have you felt the urge to drink alcohol in the past 24 hours?	• Not at all
	• Slight
	• Moderate
	• Strong
	• Very strong
	• Extremely strong
**8. Attempts to cut down and quit**	
a. How many attempts to cut down on your drinking alcohol have you made in the last 12 months (e.g. by drinking less, choosing lower strength alcohol or using smaller glasses)? Please include all attempts you have made in the last 12 months, whether or not they were successful, AND any attempt you are currently making.	
b. During your most recent attempt to restrict your alcohol consumption, was it a serious attempt to cut down on your drinking permanently?	• Yes
	• No
**9. Help sought**	
a. Which, if any, of the following did you use to help restrict your alcohol consumption during the most recent attempt?	• Any medicines (e.g., acamprosate (Campral), disulfiram (Antabuse), nalmefene (Selincro)
	• Attended one or more one-to-one or group counselling\advice\support sessions for help with drinking
	• Attended a specialist alcohol clinic or centre for help with drinking
	• Consulted a community pharmacist for help with drinking
	• Phoned a helpline for help with drinking (e.g. DrinkLine)
	• An alcohol self-help book or booklet
	• Visited a website for help with drinking
	• Used an alcohol application (‘app’) on a handheld computer (smartphone, tablet, PDA)
	• Hypnotherapy for help with drinking
	• Acupuncture for help with drinking
	• Other (please specify)
**10. Triggers of quit attempt**	
a. Which of the following, if any, do you think contributed to you making the most recent attempt?	• Advice from a doctor\health worker
	• Government TV\radio\press advert
	• A decision that drinking was too expensive
	• I knew someone else who was cutting down
	• Health problems I had at the time
	• A concern about future health problems
	• Something said by family\friends\children
	• A significant birthday or event
	• Improve my fitness
	• Help with weight loss
	• Detox (e.g., dry January)
	• Other (please specify)

Those who score 8 or more (i.e. indicating hazardous and or harmful alcohol consumption and possible dependence) on the AUDIT or 5 or more on the AUDIT-C (i.e. indicating high-risk consumption) at baseline, are then asked the following questions.

#### Current attempts and motivation to reduce alcohol consumption

One of the aims of the Department of Health’s ‘Reducing Harmful Drinking’ policy, is to encourage adults to reduce their alcohol intake to recommended levels [3]. Thus participants are asked if they are currently attempting to reduce their alcohol intake (see Table 2: Question 2) and whether they are motivated to do so (see Table 2: Question 5). Question 5 was derived from the Motivation To Stop Scale (MTSS) used in the STS, which has been shown to be a reliable predictor of attempts to quit smoking [42]. The reason for using this scale is twofold: first, insofar that it proves valid and reliable, it is useful to have a single-item measure for motivation and, secondly, the scale will allow a meaningful comparison between the relative levels of motivation for changing the two behaviours.

#### Receipt of health professional advice on drinking

Participants are asked if they received any advice about their alcohol consumption from their GP and the form of this advice i.e. whether they were given help within the surgery or referred to a specialist service (See Table 2: Question 3). It is recommended by the National Institute for Health and Care Excellence (NICE) that GPs provide adults with brief advice on their alcohol consumption, and offer the provision of self-help materials or a referral to a specialist clinic [43]. GP brief advice also forms part of the Department of Health’s ‘Reducing Harmful Drinking’ policy [3]. Previous studies have shown that GP advice is successful in reducing alcohol intake and encouraging treatment for alcohol dependence [44].

#### Types of drinks consumed

In the 2009 Omnibus Survey, ‘Drinking: Adults’ behaviour and knowledge’, women were proportionately less likely to drink beer and more likely to drink wine, fortified wine, spirits and alcopops than men. There were also significant age differences, with spirits being the most popular drink among women aged 16 to 24; in comparison with wine among women aged 45–56 years of age. To determine whether relationships exist between these and other socio-demographic variables, the ATS also assesses the type of drinks consumed by participants (See Table 2: Question 4). This is measured using a similar categorisation system to the ONS Omnibus Survey, that is, which types of alcoholic drink the respondents consume most often [45].

#### Amount spent

Previous surveys have estimated that UK households spend around £15 billion a year on alcohol; although this is likely to be an underestimation given that the Government received £18.2 billion in alcohol duty and tax in 2013/2014 [46]. Thus expenditure on alcohol accounts for around 18% of total expenditure on all food and drink [47]. Households with individuals aged 60–64 spend more on alcohol, tobacco and narcotics, than those with individuals under 30 (£16.40 each week versus to £10.50) [48]. It is important to track expenditure over time and the association with socio-demographic characteristics to help inform policy, but also assess the impact of alcohol-related interventions. Thus, the ATS also assesses the amount spent on alcohol per week (See Table 2: Question 6).

#### Urges to drink

The urges to drink question (See Table 2: Question 7) was derived from the urges to smoke question used in the STS. This has been shown to be a valid measure of the severity of cigarette dependence in terms of predicting relapse following a quit attempt [49]. Several studies using a variety of methods have also shown that urges and cravings to drink predict relapse following treatment for alcohol dependency [50]. Although items exist for assessing alcohol dependence, this 1-item measure will allow comparisons with tobacco dependence.

#### Serious attempts to reduce intake

How many serious attempts participants have made to cut down is an important measure of response to interventions (e.g. [51]) (See Table 2: Question 8). Number of attempts to reduce alcohol consumption was included as the majority of individuals will most likely opt for safer levels of drinking as opposed to complete abstinence [52].

#### Help sought and motives for recent attempts to reduce intake

Questions relating to the most recent attempt to cut down on drinking include: 1) assessing what help was sought (e.g. medication, group counselling or helpline); and 2) reasons for trying to cut down (e.g. advice from doctors, an advert or health problems) (See Table 2: Questions 9 and 10).

The most recent English policy on alcohol use stated that it intended to improve treatment of alcohol dependence [5]. Current treatments recommended by NICE include medication (e.g. acamprosate, disulfiram and naltrexone), which help to prevent relapse and/or to limit the amount of alcohol consumed; and counselling in the form of self-help groups, 12-step facilitation therapy, cognitive behavioural therapy and family therapy [53]. Evidence suggests that drugs such as acamprosate significantly reduce the risk of any drinking and increase the rate of abstinence; while there appears to be some evidence for the effectiveness of psychosocial interventions, including cognitive-behavioural coping skills training and motivational interviewing [54,55].

### Six month follow-up

At 6-months follow-up participants who score 8 or more (i.e. indicating hazardous or harmful alcohol consumption and possible dependence) on the AUDIT or 5 or more on the AUDIT-C (i.e. indicating high-risk consumption) at baseline are asked to complete: 1) the AUDIT and questions regarding their current attempts to reduce intake and motivation to cut down; 2) if they received GP advice on alcohol consumption; 3) the types of alcohol typically consumed; 4) the amount spent on alcohol; 5) strength of urges to drink; 6) how many attempts to cut down they have made in the previous 6 months; 7) how long ago their most recent serious quit attempt started and how long it lasted before they went back to drinking as before or more heavily; 8) what they used to help them cut down and what contributed to their most recent attempt; and 9) whether their attempt was planned or unplanned. A key motivation for the follow-up is to assess prospective associations between changes in alcohol consumption and exposure to real-world events and treatments [28,56]. The reason for limiting the follow-up to those who are at least classified as hazardous/harmful or high-risk drinkers at baseline is that these types of analyses among abstinent and moderate drinkers are less relevant.

### Data analysis and dissemination

Descriptive statistics will be used to describe the basic features of the data, including the socio-demographic profile of participants and occurrence of alcohol related behaviours. These will include parameter estimates (e.g. means and percentages), number of participants, and measures of spread i.e. confidence intervals and standard deviations. When reporting prevalence data, data will first be weighted using a rim (marginal) weighting technique. This involves an iterative sequence of weighting adjustments whereby separate nationally representative target profiles are set (for gender, working status, children in the household, age, social-grade and region). This process is then repeated until all variables match the specified targets.

To assess differences among groups where the outcome is normally distributed, t-tests for two groups (e.g., men and women) and ANOVA (or related methods e.g. ANCOVA) for comparisons of more than two groups (e.g. social-grades AB, C1, C2, D and E), will be used. If parametric assumptions are violated, transformations will be performed or appropriate non-parametric tests employed (e.g., Kruskal Wallis and Mann–Whitney U). For dichotomous outcomes, log-linear regression or chi-squared tests will be implemented. When adjustment for confounding variables is required, or to examine the association between a quantitative independent variable and quantitative outcome, multivariable linear regression will be used. For the association between continuous predictors and dichotomous outcomes, Generalised Linear Models will be adopted, specifically the binomial family for odds ratios or log-binomial for relative risks.

To assess trends and changes over time as a function of policies and population-based interventions, interrupted time analysis will be used as this allows adjustment for autocorrelation and consideration of underlying trends in the time series [57]. Mediation analyses, segmented regression and non-linear regression analyses, will also be used where appropriate (e.g., segmented regression when assumptions of time-series are violated and there is no evidence of autocorrelation). Mediation analysis allows researchers to assess which factors ‘mediate’ between independent and dependent variables [58]; segmented (or piecewise) regression analysis allows the independent variable to be partitioned into separate intervals when its relationship with the dependent variable changes at a ‘break point’ [59]; while non-linear (polynomial) regression can be used when relationships represent a curved line [60].

Bayes Factors will also be calculated where appropriate. These indicate the relative likelihood of a hypothesised difference/association (hypothesis 1) versus no difference/association (hypothesis 2); and allow one to distinguish between two interpretations of a null result: there is evidence for the null-hypothesis or the data are insensitive in distinguishing an effect. The latter can be rejected if the study is adequately powered, however, there are problems with this in practice. First, calculating power requires the specification of a minimally interesting value. This is often difficult, particularly when similar studies have not been conducted. Secondly, power does not use the data itself in order to determine how sensitively that very data can distinguish the null and alternative hypotheses [61].

The Bayes factor is given by:

$$ BF=\frac{P\left( Data\Big|{H}_1\right)}{P\left( Data\Big|{H}_2\right)} $$

which is the probability of the data given hypothesis 1 (*H*_1_) over the probability of the data given hypothesis 2 (*H*_2_); and thus, is simply a ratio of the likelihoods of the two hypotheses. Bayes factors vary from 0 to ∞, where 1 indicates that the data do not favour either hypothesis; values greater than 1 indicate increasing evidence for the alternative hypothesis over the null; and values less than 1 indicate increasing evidence of the null over the alternative. Jeffreys [62] suggested that ‘substantial evidence’ be reflected by a factor less than 1/3 or greater than 3, with any value between these being only ‘anecdotal’ evidence.

Results will be disseminated using a website (www.alcoholinengland.info) and regular updates to key English stakeholders, including Public Health England (PHE) and the Department of Health policy and communications teams. All publications will be discussed in advance with the ATS Study Advisory Group. This group was appointed to oversee and input strategically on the overall progress, priorities and planned analyses of the ATS.

## Discussion

The ATS has several important strengths, including the ability to examine changes in the prevalence of harmful drinking and other key performance indicators, such as attempts and motivation to cut down, in a timely manner. Tracking monthly changes permits a much more sensitive test of the possible effects of interventions than can be achieved by annual national surveys. It will also provide information on methods of reduction and how these relate to success rates and contextual variables, such as socio-demographic characteristics. The large sample size and follow-up will also allow for relationships between government policies/alcohol initiatives and reductions in consumption to be accurately estimated and tested prospectively. The data will provide quick and direct estimates of policy impacts on consumption and also inform the estimation of longer-term health benefits and cost saving by providing evidence to incorporate into existing policy appraisal models [63]. The addition of the ATS to the STS will also allow comparisons to be made between tobacco and alcohol use.

Although the ATS is restricted to data from England and cannot document the situation for the rest of the UK or other countries; it provides a framework on which other national surveys can be modelled. Moreover, given that alcohol policies and treatment in England are similar to other countries, findings from the ATS, to some extent, can be used to inform international policy [64].

The main limitation, as with all survey data, is the likelihood of underreporting alcohol intake. This is often due to a combination of poor recall, participants being untruthful, inadequacies in measurement instruments and sampling bias [65,66]. The extent of underreporting is somewhat mitigated by the use of computer assisted interviews since the presence of a computer enhances participants’ perceptions of privacy and thus increases responses to sensitive questions [67]. Recall bias is also limited in the ATS by assessing typical alcohol consumption; and including response categories (which act as triggers). The ATS also affords the ability to cross-validate responses due to the inclusion of questions measuring similar underlying constructs. For example, answers to the AUDIT are likely to be analogous to measures of urges to drink, while motivation to quit is likely to be analogous to attempts to reduce intake.
